# Standards for evaluating the quality of undergraduate nursing e-learning programme in low- and middle-income countries: a modified Delphi study

**DOI:** 10.1186/s12912-023-01235-7

**Published:** 2023-03-20

**Authors:** Moses Mutua Mulu, Champion N. Nyoni

**Affiliations:** School of Nursing, Faculty of Health Sciences, University of the Free State, Bloemfontein, South Africa

**Keywords:** Online learning, Experts, Nursing, Quality, Modified Delphi, Standards, Low and middle-income countries (LMICs)

## Abstract

**Background:**

The lack of standards for evaluating the quality of undergraduate nursing programmes hampers the evaluation of e-learning programmes in low- and middle-income countries. Fragmented approaches to evaluation coupled with a lack of uniform criteria have been a major deterrent to the growth of e-learning. Adopting standards from high-income countries has contextual challenges in low- and middle-income countries (LMICs). Holistic approaches coupled with uniform standards provide information to stakeholders hence the quality of the programmes is measurable. The e-learning situation in low-and middle-income countries provided an impetus to develop and validate these standards.

**Design:**

A modified Delphi technique.

**Review methods:**

Fourteen experts with experience and expertise in e-learning and regulation of undergraduate nursing from fourteen countries from LMICs participated in three rounds of the modified Delphi process. A pre-described set of standards was shared electronically for independent and blinded ratings. An 80% threshold was set for consensus decisions. The standards were modified based on experts’ comments, and two subsequent rounds were used to refine the standards and criteria.

**Results:**

At the end of round one, the expert consensus was to keep 67, modify 39 and remove three criteria. At the end of the second round, the consensus was to modify 38 and remove one criteria. In the third round, experts agreed that the standards were feasible, usable, and practical in LMICs. A total of six broad standards with 104 criteria were developed.

**Conclusion:**

The Technological bloom permeating all spheres of society, including education is an essential component in the development of e-learning programmes. E-learning in nursing education requires critical evaluation to ensure quality in undergraduate nursing programmes. The intricacies of the Low and middle-income context were taken into consideration in developing the standards to offer sustainable evaluation of the quality of e-learning in LMICs, and local solutions to local problems.

**Supplementary Information:**

The online version contains supplementary material available at 10.1186/s12912-023-01235-7.

## Introduction

The mere availability of nurses is insufficient to meet the global needs for high-quality nursing staff to respond effectively to current health challenges [1]. They in fact also need the required competencies and must be effectively trained to meet the health needs of communities [2]. There is an increased demand for high-quality degreed nurses with improved skill sets across various contexts, including low- and middle-income settings [3]. A range of evidence supports Choi et al. (2021); Porat- Dahlerbruch et al. (2022); Drasiku et al. (2021) and Harrison et al. (2019) [4–7], nurses with a degree-level qualification offer better nursing care and improved patient health outcomes, including reduced preventable hospital deaths. Therefore, numerous strategies are used to accelerate the upgrading of nurses from diploma or associate degree qualifications to bachelor’s degrees [8]. Most of these upgrading programmes are offered through e-learning or open distance learning approaches, taking advantage of the nurses’ prior knowledge and skills [9]. The flexibility provided by e-learning allows student nurses to navigate various competing interests such as work, family, and professional development [10].

E-learning has challenged conventional teaching approaches and revolutionised learning with ease and accessibility otherwise unimagined in education [11]. E-learning is an approach to facilitate the application of knowledge and information and enhance learning using personal computers, CD-ROMs’ and the internet for education [12]. The critical components of an e-learning programme are divided into four broad categories: curriculum, learners, educators, and, support, and evaluation (Misut & Pribilova, 2014; Gautam & Tiwari, 2016) [13, 14]. Nursing education curricula have also been significantly influenced by technological advances Risling, (2017) and will be shaped by e-learning in the future as the technological landscape is growing with advances in all spheres of human interaction [15]. Murebwanyire et al. (2015); Harerimana & Mtshali (2020) describe how e-learning was identified as a large-scale method to upgrade nurses to a diploma and higher diplomas [16, 17]. The demand for nurses with a degree has resulted in unprecedented increase in the number of nurses upgrading qualifications from diploma to degree. This gap has been met by e-learning, enabling many nurses to access opportunities for learning [18]. E-learning has been instrumental in developing the capacity for an alternative learning environment for continuing professional development and lifelong learning, especially for those whose work schedules offer limited time to travel over long distances to acquire knowledge [19, 20]. Regmi and Jones (2020) state that the ability of e-learning to transcend barriers and enhance learning has modelled it to be a mainstream approach in health sciences [21].

The shifting dynamics of the learner, educator, and environment have led to unprecedented and transformative approaches to teaching and redefined nursing education, thus shaping e-learning [22, 23]. Using e-learning programmes, therefore, provides appropriate learning experiences that can support clinical learning experiences with demonstratable teamwork, leadership, practical knowledge, and an emphasis on work readiness [24]. The delicate balance between what constitutes a successful e-learning programme and quality evaluation in undergraduate nursing programmes provides an avenue for deliberations and developing quality assessment tools applicable in LMICs.

Africa is experiencing significant growth in e-learning, especially since the COVID-19 pandemic, even though e-learning is still in its infancy in many LMICs [25, 26]. The proliferation of internet usage in Africa is dissimilar to undergraduate learners who consider e-learning as a mode of study; whilst the former figures are surging greatly, the latter is slowly gaining familiarity among undergraduate learners in LMICs [27]. E-learning in Africa developed in 1996 with centres in Ethiopia, Ghana, Kenya, Uganda, and Zimbabwe. The initial programmes were on science, engineering, and business [27]. In Kenya e-learning programmes in nursing education were offered through a distance learning model using textbooks, as a computer-based approach to e-learning was a challenge [28]. However, there was no public and institutional policy and standards to support e-learning initiatives in most LMICs during those early days. This lack of support was further complicated by a lack of infrastructure, poor internet proliferation, a lack of capacity among institutions, educators and learners, limited familiarity with technology in education and a negative attitude to e-learning [29, 30].

Standards offer a yardstick for evaluating the quality of an educational programme. Thus, e-learning programmes must be evaluated to determine their quality [31]. Standards in e-learning are documented agreements containing technical specifications including rules, guidelines, and definitions of characteristics to ensure that materials, products, processes, and services are fit for their purpose with specific parameters in order to ensure that e-learning courses are apt and useful for their user [32]. Quality standards for assessing e-learning programmes should be anticipated while developing the programme to provide methodological, valid, and objective assessment criteria [33]. Delva (2019) describes that standards provide a roadmap to assess the quality of e-learning [34]. The quality of an e-learning programme includes learners who are ready to learn and supported to learn, environments with adequate resources, content reflected in relevant curricula for the acquisition of relevant skills, educators able to carry out roles in skill acquisition, assessment and outcomes that demonstrate knowledge, effective use of ICT by learners and educators, and skills and attitudes linked to desired results [21, 35, 36]. Standards for evaluating the quality of undergraduate e-learning programmes can be adapted for nursing education and broadened to include clinical components. These standards could, therefore, redefine how quality in nursing education should be evaluated specifically in LMICs [21, 37]. Standards in use in LMICs are adopted from High income countries and these offer contextual challenges in implementation [9]. Focus should be on context specific standards developed in LMICs.

Evaluating the effectiveness of e-learning programmes can guide the development of nursing education in order to determine its value and importance. This can be best done by making use of a set of standards. This set of standards will inform stakeholders on the excellence of these programmes [38, 39]. As e-learning programmes develop, so does the need to monitor the quality of e-learning and its components to provide an effective programme. However, the evaluation of e-learning should not consider constituent parts only, but a holistic evaluation of the e-learning programme [40]. A common pitfall of most evaluations in LMICs is the lack of uniform criteria for evaluating the quality of the e-learning programmes, coupled with the lack of standards.

This has led to the inability of regulatory bodies to evaluate such programmes in nursing education [40]. The lack of holistic standards has been a stumbling block in establishing quality evaluation tools in e-learning programmes in LMICs, as most evaluations focus more on the components of e-learning than on the entire programme collectively.

The use of fragmented approaches to assess e-learning in LMICs is rooted in an over-emphasis on specific areas of e-learning rather than on the programme as a whole. This fragmentation is attributed to a lack of standards [29, 41, 42]. Barteit et al. (2020) further identified that most evaluations of e-learning in LMICs are small-scale, and more rigorous evaluation methods are required to understand the strengths and shortcomings of e-learning programmes [29]. Baker et al. (2021) describe that standards provide a clear delineation of the requirements of programmes to meet the global needs of high-quality nursing personnel able to respond effectively to current health challenges [43]. Therefore, the lack of direct standards for evaluating e-learning programmes across LMICs can explain the intransigence of evaluating these programmes.

The need for context-specific standards for e-learning to meet the specific needs of the programme whilst matching with international standards of quality is required in LMICs [41, 44]. The paucity of standard evaluation tools for e-learning in LMICs limits the validity of evaluation results [29, 41]. However, e-learning can be evaluated against established standards which already exist and might not be specific to LMICs [34]. We contend that adopting evaluation criteria from developed countries to be applied in LMICs offers context-specific challenges to the development of e-learning. The adopted evaluation criteria, albeit contextually sound in the developed country, could mollify other features that may potentially inform more robust e-learning systems in LMIC contexts. The novelty of these developed and validated standards in LMICs, keeping in mind the contextual challenges compounding LMICs, offers feasible, usable, and practical techniques to holistically evaluate the quality of undergraduate e-learning nursing programmes.

## Methods

### Aim

This paper reports on a consensus study on standards for evaluating the quality of undergraduate nursing e-learning programmes in LMICs.

### Design

This paper is drawn from an overarching project which sought to develop, validate, and test standards for evaluating the quality of undergraduate nursing e-learning programmes in Kenya. Preliminary work was conducted in an earlier phase of the study through an integrative review which identified six broad thematic areas with a total of 109 criteria for consideration (see Additional file 1). The standards were divided into six categories: curriculum planning, proficiency of the educator, learner proficiency and attitude, infrastructure for learning, support, and evaluation. Each of the standards had different criteria. The modified Delphi is similar to the full Delphi in respect to intent and procedure. The modification consists of beginning the process with standards developed from integrative reviews [45]. The modified Delphi has additional benefits of reduced bias, anonymity, and controlled feedback to participants [45, 46]. This study applied a modified Delphi technique used to achieve consensus on standards or quality indicators, especially in areas with no recommended guidelines for a specific problem [47, 48]. For a modified Delphi to be successful and maintain quality, it was important to identify the problem, meticulously select the panel of experts, ensure the anonymity of experts, obtain controlled feedback, conduct iterative Delphi rounds, gain consensus on the criteria, analysis of the consensus, closing criteria and result stability [49]. In line with Brown and Crookes (2016), equal anonymous contributions for minimising power differentials and reducing bias were applied in the modified Delphi [50]. Consensus-building was developed by:

- applying the three steps of identifying potential experts for inclusion in the expert panel,
- conducting a modified Delphi and.
- presentation of the final standards.

#### Step 1: Identifying the experts to include in the panel

Experts were selected based on the following inclusion criteria: experience in e-learning in LMICs through teaching and/or policy formulation related to e-learning, recent publication history in e-learning in LMICs, a nursing or health sciences background and technical knowledge of e-learning. This selection involved administrators, digital campus, and e-learning support personnel from a number of LMICs.

The inclusion criteria were used in the selection process to ensure that objectivity was maintained throughout the process.

Firstly, the authors from the studies identified in the integrative review stage were included. The total number of experts in this first phase was ten. Secondly, both authors identified individuals within their networks who met the inclusion criteria. The total of 15 experts identified were composed of specialists in nursing education from Africa and Asia. These 34 identified experts were invited to be part of the study through an email with a participation information leaflet and a consent form.

The experts were also requested to nominate other experts who met the inclusion criteria, and these nominated experts were also invited participate in the study. The 14 experts who volunteered were included in the modified Delphi.

#### Step 2: Data collection

The data collection tool for the first round was developed using the REDCap application ® and Survey Monkey ®. A total of six standards with a total of 109 criteria were used. The modified Delphi had three rounds of ratings, reviews, and reflections by the experts over sixteen weeks between May and August 2022.

#### Modified Delphi round 1

Round 1 of the modified Delphi had an online questionnaire, The experts were presented with all six standards and 109 criteria and asked to state whether they agreed, disagreed or were not sure about a stated criterion. We used 3-point Likert scale questions and additional space for the experts to provide their opinions on the standards in their own words, this would assist in a clear interpretation and actionable results for the next phase of the study. Experts had two weeks to complete the questionnaire. Fourteen responses were received, translating to a 100% response rate. The consensus was set at 80%, with areas of disagreement providing an opportunity for a second round [46, 51]. Responses to the modified Delphi, phase 1, were analysed by the authors over two weeks. Any question reaching the consensus threshold was regarded as finalised and was not included in the second round. In cases where at least 80% consensus was not reached by the experts, if they were unsure about a standard and-or were unsure about a standard and or its criterion, the standard and its criterion were reformulated based on the feedback from this round and included in the questionnaires for the second round.

#### Modified Delphi round 2

The questionnaire for the second round required the experts to review the standards that did not reach the consensus threshold. They could either keep or, discard standards and criteria or suggest alternatives or modifications. The standards were presented in RED Cap format with a similar 3-point Likert scale approach. At the end of the second round, the standards proposed by the experts were deliberated, the decisions to retain or remove were made in line with the expert opinion, and the final standards were developed. The consensus reached in the second phase was considered final.

#### Modified Delphi round 3

This final round aimed to provide the experts with the finalised standards after the iterative process. Experts were requested to comment on their feasibility, usability, and practicality in LMICs. The experts agreed on all aspects.

### Data analysis

Data from the modified Delphi rounds were analysed quantitatively by descriptive statistics and qualitatively by themantic analysis.

Data analysis involved analysing each criterion for consensus [52]. After each rating round, analysis was done on each standard, and those standards with a consensus of 80% across all the responses were moved to the final draft. The standards and criteria that did not reach 80% consensus and areas of disagreement were rerated in the second round, and the recommendations and comments made by experts were considered. Responses from the second round provided ratings with areas of agreement with modifications, and the standards moved to the final draft.

All areas of agreement from the second round were incorporated, and those that did not reach the threshold were eliminated from the standards. The third round presented the final standards through the iterative process.

### Rigour

#### Ethical approval

was sort from the Health Sciences Research Ethics Committee (HSREC) and National Commission for Science and Technology (NACOSTI). Trustworthiness reflects the rigour of a study and concerns ensuring its credibility, dependability, confirmability, and transferability [53]. The initial findings of the study were discussed to identify opinions and feedback and to determine dependability The views of experts were sought who are leaders in nursing education and e-learning and are familiar with the evaluation of e-learning. Rigour was thus maintained by selecting a heterogeneous sample of experts with different backgrounds but relevant experience and the capacity to validate expert opinion [54]. All activities were documented to determine the confirmability of the findings, and a report of the process was prepared. The results were shared amongst individuals of similar situations and experiences (LMICs) to determine transferability, and the results were confirmed to be feasible and useable. A blinded review process ensured that experts’ identities were hidden to avoid group bias [54]. Rigour is further maintained by consistency and meticulous adherence to the process [54].

## Results

Most of the participating experts from Africa and Asia were female n = 8 (57%) with roles in nursing education regulation, directors of e-learning, nursing, and midwifery education. The male participants n = 6 (43%) had roles in nursing and midwifery education, health professions education backgrounds in nursing education and e-learning (see Table 1.)

**Table 1:** Demographic characteristics of experts

Gender	
Male	6 (43%)
Female	8 (57%)
Continents of residence	
Africa	10 (71%)
Asia	4 (29%)
Educational qualification	
PhD	12(86%)
Masters	2(14%)
Roles of experts	
Nursing education regulation	1 (0.07%)
Director of e-learning	1 (0.07%)
Nursing education	6 (43%)
Midwifery education	2 (14%)
Health professions research education	3 (21%)
E-learning	1(0.07%)

**Fig. 1:**
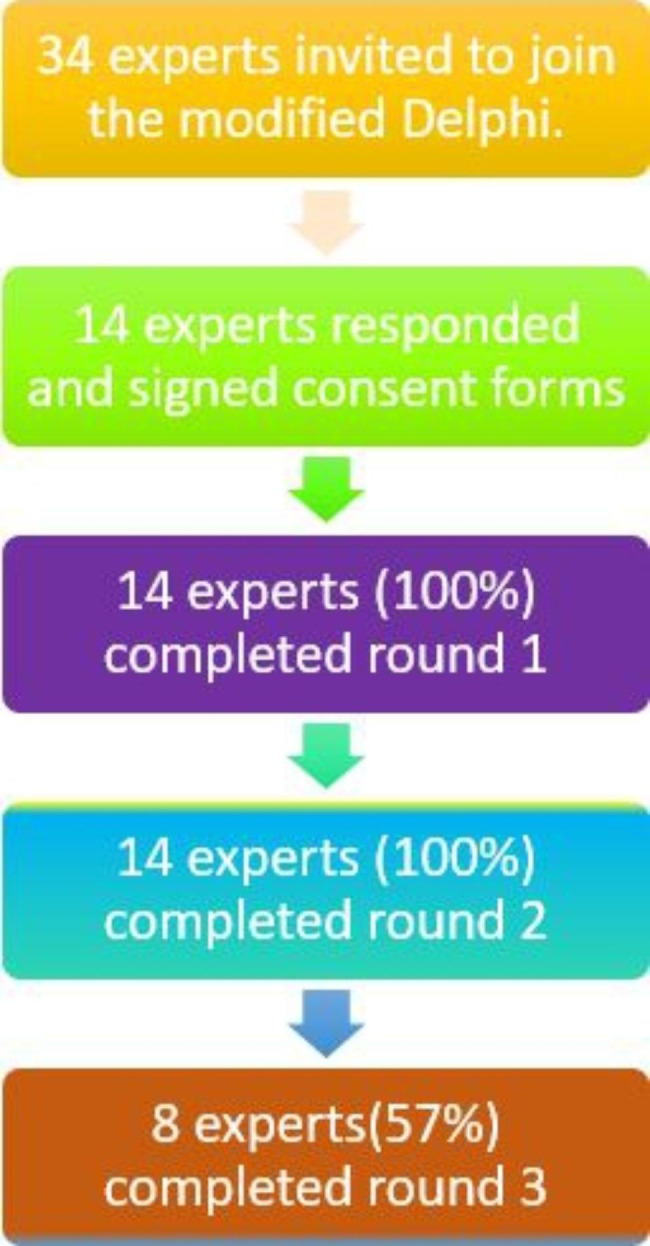
Expert flow throughout the study

A total of 14 experts consented to participate (see Fig. 1).

**Fig. 2:**
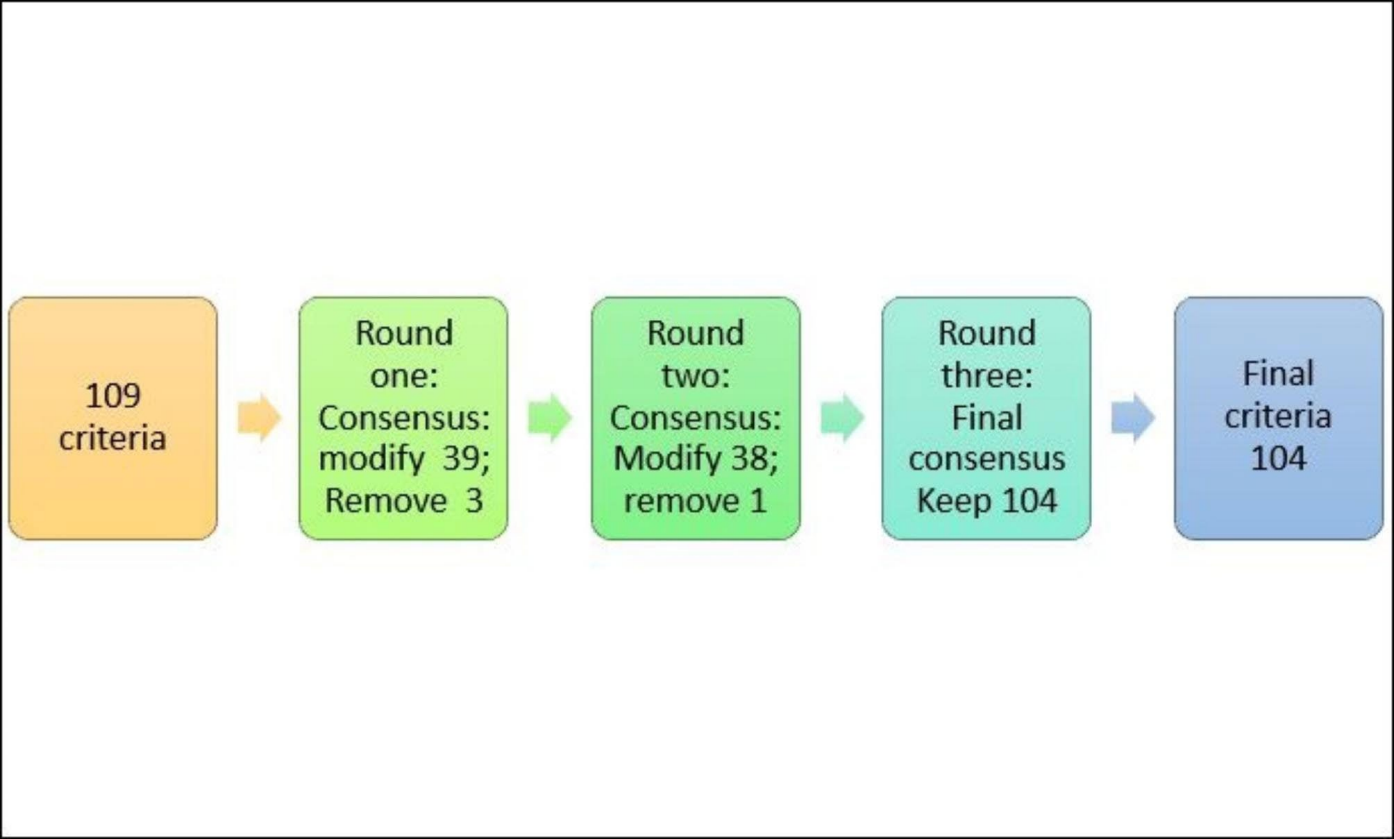
The flow of standards throughout the process

A total of 109 criteria were presented to the experts at the start of the modified Delphi process. At the end of round 1, experts agreed to keep 67, modify 39 and remove three criteria. The 67 criteria were included in the final standards and were not entered into the second round. The remaining 39 criteria were amended based on the experts’ choice and proceeded to the second round. At the end of the second round, the expert panel reviewed 39 criteria from the six different domains and either agreed, or disagreed, with the criteria and had the option to offer alternative criteria. The panel suggested the modification of 38 criteria and removal of one. The expert panel reached consensus on all the presented standards, and a final draft was developed. The third stage was the presentation of the final standards to the experts for final approval. A total of eight (57%) experts responded to the final survey and they were required to reflect on the usability, feasibility, and practicality of the standards. The consensus on the three aspects was 100% (see Fig. 2).

In the modified Delphi process, 104 standards across six domains were developed. The feasibility, usability, and practicability across LMICs were at 100%, with the experts agreeing that the standards were applicable in LMICs (see Table 2).

**Table 2:** Summary of standards through the stages of the study

Standards		Round 1		Round 2				Round 3			
		Total criteria	Criteria with consensus		Total criteria	Criteria with consensus	Criteria removed		Total criteria	Feasibility, usability & practicality	
Curriculum planning		30	21 (70%)		30	29 (96%)	0		30	8 (100%)	
Proficiency of educator		20	9 (45%)		19	19 (100%)	0		19	8(100%)	
Learner proficiency and attitude		14	11 (78%)		14	11 (78%)	3		14	8(100%)	
Infrastructure for Learning		17	14 (82%)		18	18 (100%)	3		18	8(100%)	
Support		15	11 (73%)		13	13 (100%)	2		13	8 (100%)	
Evaluation		13	10 (83%)		12	12 (100%)	2		10	8 (100%)	

The standards were divided into six categories namely: curriculum planning, proficiency of the educator, learner proficiency and attitude, infrastructure for learning, support, and evaluation. Each of the standards had different criteria (see additional file 1 shows this in more detail).

## Discussion

The success of e-learning in high-income countries is anchored on a combination of models such as Delone and McLean’s information systems success model, user satisfaction models and technology acceptance models [55, 56]. Certain challenges were reported regarding the model of Delone and Mclean (2003) due to complexity for implementors [57]. Several revisions were made to the model with evident integration challenges. The technology acceptance models have been widely used to evaluate e-learning success. However, criticism has been directed towards the application of these models and attempts to expand these models leading to theoretical challenges and confusion [58]. User satisfaction models, focusing on learner satisfaction have been extensively used in various disciplines, including health sciences [59]. The standards developed in this study differ extensively from those readily utilized in high-income countries. Despite this, the authors’ specific focus was the LMIC context, which differs immensely in terms of curriculum, availability of resources, contextual culture, infrastructure, educators, learners, and technical support. A plethora of models are available for evaluating e-learning quality in high-income countries with comprehensive criteria that are successful in that context. The standards developed in high-income countries, are applicable in contextually similar settings and have been challenging to apply in LMICs [60]. This is the essential focus behind the development of these standards namely to provide quality standards for evaluating undergraduate nursing programmes in LMICs and involvement of experts within LMICs.

The urgency to develop standards for e-learning in LMICs is anchored in its rapid expansion, especially post-pandemic, and the need to maintain quality in the education of nurses who are critical determinants of health outcomes in communities and the health of the patients they serve [61]. Currently, there are no standards for evaluating the e-learning quality in LMICs. The central focus in developing such standards was to bridge a gap and develop a holistic evaluation of e-learning, as opposed to a fragmented approach model [36]. The focus was on highlighting the challenges regarding e-learning in LMICs, the available opportunities, and best practices across various LMIC settings [62–64]. These perspectives shaped the foundation of the study and grounded the process of developing e-learning standards in LMICs. This approach was pragmatic in developing the quality of e-learning, especially in LMICs which are beset by a host of challenges. It offers a systematic perspective for university boards of management approach regarding the needs of e-learning rather than a fragmented perspective which obscures the development of e-learning [65, 66]. Widyanti et al. (2020) found that most university management structures realized the value of e-learning educators when the COVID-19 pandemic led to the global closure of universities [67]. This led to a higher cognitive workload in e-learning compared to face-to-face learning, coupled with the weight of designing content, teaching, evaluating learners, and infrastructural challenges [66]. The ensuing confusion led to some universities opting to close indefinitely, while those that managed to remain open had difficulty manoeuvring the e-learning landscape with no standards to guide the transition process [68]. The timing of these standards is interwoven with the desire of most universities to actualise e-learning as a mode of study. It is quickly becoming a reality in the health science departments and the quality of the programmes is critical.

The current study reports the findings of a modified Delphi on standards for evaluating the quality of undergraduate e-learning programmes. The study accentuates expert opinion and consensus on the standards in LMICs and highlights various spheres of concern to experts across the different criteria. Shifting learning practices and the advent of e-learning and virtual learning are shaping contemporary approaches to understanding the curriculum [69]. The curricula in use were developed in previous years and were subject-centred. While it was essential at the time, the shift to learner-centred approaches in e-learning demands a change in the approach to curriculum design [70, 71]. The two interacting constructs of societal needs and learners are critical factors in shaping a curriculum. The former is the foundation of the traditional curriculum, while the latter shapes the e-learning curriculum [69]. Integrating technology into the curriculum, blending thinking, innovation, and ICT skills is inevitable in engaging the millennial learner who is central to the organisation of learning whilst the educator supports learning. The use of a traditional face-to-face curriculum in e-learning has therefore been a huge impediment to the growth of e-learning, and undermines the progress made toward improving e-learning quality in LMICs [72]. A curriculum specific to e-learning is a starting point for quality undergraduate e-learning programmes, as the curriculum would provide all stakeholders with a measurable structural plan for the programme’s accountability and social responsibility, especially in LMIC settings [69, 73]. Furthermore, the novelty, clarity and comprehensiveness of these standards will enable the development of an e-learning curriculum. We argue that with the shifting dynamics of the learners, content, teaching approaches and demands of modern-day education, a rigid traditional face-to-face curriculum would be impractical in e-learning.

The second standard regarding the proficiency of educators in is a common but often overlooked concept in e-learning. The following aspects are important: the attitude of the educator; learners who consider educators as role models, and the educators’ attitude toward e-learning that rigorously affects the learners’ attitude [59, 73]. Experts agree that the increased workload associated with e-learning, class size issues, teaching resource availability, mental workload and educator motivation are critical factors in educators’ perceptions of e-learning [74, 75]. Almahasees et al. (2021) identified differences in training and e-learning delivery between high-income and low-income countries. The lack of teaching resources available to educators, reduced knowledge of ICT and the challenges of class control emerged as areas of agreement among most experts [76]. This has been a problem in the development of e-learning in low-income countries [26, 74, 77]. The educators’ motivation for the use of e-learning, teaching styles, their perception of e-learning and control of technology will shape the behaviour of learners and significantly improve learner satisfaction [78, 79]. The experts agreed on the use of e-learning to advance the continuing professional development (CPD) of educators to ensure a commitment to continuously improve their knowledge and skills. This is supported by Mlambo et al. (2021) who found that the accessibility and availability of CPD through e-learning for educators greatly improved their competence in the use of e-learning tools [80]. The role played by educators in shaping learners’ perceptions cannot be overestimated, especially in e-learning.

The third standard, the relationship between learner proficiency and attitude and the use of e-learning and consequent success in e-learning is significant and leads to the satisfaction of learners [55, 81]. Perceived satisfaction of learners regarding the use of e-learning is an essential measure of the success of e-learning and will lead to the optimal use of the system [82]. Usefulness is a key determinant of the success of e-learning among learners, because if they perceive e-learning to be useful, it will increase to the likelihood of their use of e-learning and significantly influence satisfaction [82, 83]. The ability of learners to objectively evaluate teaching and learning was an area of concern as most of the learners were not actively involved in the evaluation of e-learning programmes compared with learner challenges limiting the objective evaluation of e-learning. However, this contrasts with Al-Fraihat et al. (2021); Penna and Stara (2007), who identified that learner-centred evaluation is essential to the quality of e-learning [55, 84]. Experts agreed that learners should be prepared in advance for e-learning and notified on how to navigate the course and its expectations. The developed standards focus on the learner preparedness, equipment, and evaluation of e-learning to ensure satisfaction and actualise the maximum benefits. Furthermore, sufficient, concise, and clear information contributes immensely to satisfaction.

Fourthly, the myriad of infrastructural e-learning challenges faced by universities in LMICs requires contextually sound standards and comprehensive evaluation, although progress in this regard is evident [85]. Policy issues surrounding e-learning infrastructure were an area of contention as most of the experts agreed that operationalising policies in e-learning is challenging in LMICs. Commitment by key stakeholders is often tenuous. This view was similarly shared by Kibuku et al. (2020) and Mbugua (2014), who identified that policy gaps have hampered higher education in the Kenyan context [74, 86, 87]. The impetus to develop these standards with a focus on policy is to ensure that stakeholder commitment is captured from the start when developing e-learning programmes. The availability of infrastructure to support e-learning was described as challenging for most LMICs, with funding and systemic challenges overshadowing meaningful progress to improve e-learning. Despite the promising internet penetration in LMICs, especially in Africa, the concentration is mostly in urban areas. In addition, the cost of internet access is prohibitive [85]. The learning management systems (LMS) in most developed countries are customised to suit their specific needs, but in LMICs, reliance on open source LMS limits the ability to customisable options [87–89]. The ease of use, system quality and satisfaction of learning management systems (LMS) by educators and learners are critical in improving e-learning [88, 89]. We argue for the commitment of governments and university management boards to strengthen the resolve toward improving e-learning which has enormous potential to scale up health workers.

Fifthly, support systems for educators and learners were an area of concern. In most LMICs, there’s is not adequate e-learning support for educators and learners [90]. Wang et al. (2018) identified that support for e-learning resources is an area of interest in e-learning [91]. Experts agreed that e-learning support is a critical and essential standard for evaluating e-learning, and its accessibility is crucial for learners’ satisfaction. Experts agree that e-learning support is essential to the operation of e-learning. There is a need to improve internet bandwidth, although better bandwidth doesn’t necessarily mean that learners and educators will use this for learning purposes. The need for control of content and site restriction is crucial in ensuring appropriate utilisation. According to the findings of Chanboualapha and Islam (2012), who compared the relationship between internet usage and learning and found that the internet has a positive relationship with learners’ knowledge [92]. The absence of social media in their study can be attributed when the study was conducted. However, Waweru (2018) found that learners spend most of their time on social media, during the time which they could be studying, leading to poor academic performance academically [93]. The site access restriction should be so that learning is not impaired. These are some of the areas in which the panel of experts expressed concern, and it was worth noting that the changes to the standards improved the quality and applications to LMICs.

Lastly, these standards will be critical for evaluating the quality of undergraduate e-learning nursing programmes. They will also assist in shaping the intricate capacity to develop local solutions to local problems in LMICs [23]. Based on the iterative process, the experts reached consensus on six broad categories: curriculum planning, proficiency of the educator, learner proficiency and attitude, infrastructure for learning, support, and evaluation, with 104 criteria for evaluating the quality of e-learning in undergraduate nursing programmes in LMICs. These standards will provide schools of nursing seeking to evaluate the quality of e-learning with a guide to practical evaluation. The standards are comprehensive and broader covering wider range of criteria than those available in developed countries. They align with nursing education at a degree level. Consequently, educators and learners will have better learning experiences with e-learning leading to quality in e-learning in nursing education hence improving patient outcomes. The feasibility, usability, and practicability of the standards’ are crucial in ensuring they are easily usable and practical for the context-specific challenges in LMICs. The complete agreement (100%) by the experts (100%) on the final standards’ feasibility, usability, and practicability is critical in applying these standards in LMICs. The crux of this study is standards for quality evaluation. The feasibility, usability, and practicality of these standards have a high propensity to improve the quality of e-learning holistically in LMICs.

### Limitations

Despite numerous efforts to recruit more experts from more LMICs, this did not materialise. This hesitation to volunteer may be due to busy work schedules and unavailability and can be explained by the critical roles of most of the experts identified in nursing education and e-learning. A further limitation was that the experts drawn from different LMICs had to be proficient speak in English.

## Conclusion

The opportunities provided by e-learning outweigh the challenges as it presents no geographic barriers, flexibility, while accommodating flexibility, creativity and critical thinking. It utilised online resources, offers an effective approach to transferring clinical knowledge, and improves the teaching experience [22, 94, 95]. However, many challenges impact the implementation of e-learning in LMICs. The salient challenges include the lack of a relevant curriculum, lack of infrastructure, lack of ICT knowledge, weakness of content development, educators and learners’ culture and lack of regular online training and seminars for educators and students to support the application of e-learning [94, 96] The rather inconspicuous challenges affecting e-learning are financial, managerial, insufficient professional development, copyright issues, and stakeholder motivation[46, 62, 97, 98]. This last aspect is rarely addressed but has an immense impact on the growth of e-learning in LMICs [63, 64, 97, 98]. Critical reflection of these challenges towards developing sustainable solutions for e-learning is vital in improving the quality of e-learning in LMICs. The standards are essential to evaluating the quality of undergraduate nursing programmes. The identification of these standards culminated in an iterative process with experts drawn from various LMICs with expertise in e-learning and quality assurance and an understanding of the challenges in the field.

The quality of an e-learning programme is evaluated using standards. Undergraduate nursing programmes have a theoretical and clinical component that further complicates such evaluation. The modified Delphi reported in this article refined standards for evaluating the quality of undergraduate nursing e-learning programmes in LMICs. The final product consisted of six standards with 104 criteria. The six standards identified were curriculum planning, proficiency of the educator, learner proficiency and attitude, infrastructure for learning, support and evaluation. These offer critical domains in evaluating the quality of undergraduate nursing programmes, and these are essential areas when evaluating the quality of e-learning. The intricacies of the LMIC context require a thorough understanding of the challenges to meticulously develop feasible, usable, and practical standards. These standards further demonstrate adaptability in low-resource settings. The standards developed are recommended for use in LMICs to evaluate the quality of undergraduate e-learning programmes. Additionally, the standards provide researchers with a basis for further research, with the evolving technological landscape, it will be thought-provoking to see the transitional changes that these standards take over time. Therefore, these developed standards are contextualised to offer sustainable evaluation of the quality of e-learning in LMICs as well as local solutions to local problems. The next steps for these standards are piloting in a local university for feasibility, usability and practicality.

## Electronic supplementary material

Below is the link to the electronic supplementary material.

Supplementary Material 1

## Data Availability

All data generated or analysed during this study are included in this published article [and its supplementary information files].

## List of Abbreviations

COVI9-19: Corona Virus 2019
CPD: Continuous Professional Development
ICT: Information, Communication, and Technology
LMS: Learning ManagementSsystem
LMIC: Low- and Middle-Income Countries
